# Thyroid‐stimulating hormone is an independent risk factor of non‐alcoholic fatty liver disease

**DOI:** 10.1002/jgh3.12264

**Published:** 2019-10-01

**Authors:** Kazuki Tahara, Takemi Akahane, Tadashi Namisaki, Kei Moriya, Hideto Kawaratani, Kosuke Kaji, Hiroaki Takaya, Yasuhiko Sawada, Naotaka Shimozato, Shinya Sato, Soichiro Saikawa, Keisuke Nakanishi, Takuya Kubo, Yukihisa Fujinaga, Masanori Furukawa, Koh Kitagawa, Takahiro Ozutsumi, Yuuki Tsuji, Daisuke Kaya, Hiroyuki Ogawa, Hirotetsu Takagi, Koji Ishida, Akira Mitoro, Hitoshi Yoshiji

**Affiliations:** ^1^ Third Department of Internal Medicine Nara Medical University Kashihara Japan

**Keywords:** hypothyroidism, liver fibrosis, non‐alcoholic fatty liver disease, thyroid‐stimulating hormone

## Abstract

**Background and Aim:**

Hypothyroidism might play a crucial role in the pathogenesis of non‐alcoholic fatty liver disease (NAFLD). The association of subclinical hypothyroidism with NAFLD has been inconsistent. The relationship of NAFLD with thyroid function parameters and subclinical hypothyroidism was determined.

**Methods:**

This cross‐sectional study included 70 patients with subclinical hypothyroidism and 70 controls with euthyroidism matched according to gender, age, and body mass index (BMI). NAFLD was diagnosed via abdominal ultrasonography. The association between NAFLD and subclinical hypothyroidism was analyzed.

**Results:**

The prevalence of NAFLD was significantly higher in patients with subclinical hypothyroidism than in those with euthyroidism. Multivariate analysis showed that subclinical hypothyroidism was an independent risk factor of NAFLD adjusted by metabolic‐related factors, such as BMI, triglyceride, high‐density lipoprotein‐cholesterol, hypertension, and diabetes. Thyroid‐stimulating hormone (TSH) was an independent risk factor of NAFLD adjusted by the same metabolic‐related factors, but free thyroxine (FT4) was not a risk factor. The FIB‐4 index, a noninvasive marker of liver fibrosis was significantly higher in patients with subclinical hypothyroidism than in those with euthyroidism. Compared with patients with euthyroidism, the proportion of the FIB‐4 index ≥2.67 was significantly higher, and the proportion of the FIB‐4 index <1.30 was lower in patients with subclinical hypothyroidism.

**Conclusions:**

TSH elevation even within the euthyroid range is an independent risk factor of NAFLD and may influence the progression of liver fibrosis, even with a normal FT4 level.

## Graphical abstract

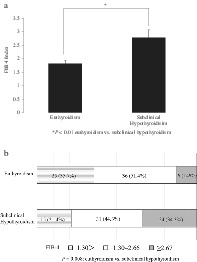

Thyroid‐stimulating hormone elevation, even within the euthyroid range, is an independent risk factor of non‐alcoholic fatty liver disease and may have an influence on the progression of liver fibrosis, even with a normal free T4 level.

## Introduction

Non‐alcoholic fatty liver disease (NAFLD) is one of the most common chronic liver diseases.^1^ NAFLD includes a broad range of conditions, such as simple steatosis and non‐alcoholic steatohepatitis, which could progress to cirrhosis and hepatocellular carcinoma. In addition, NAFLD can increase the incidence of cardiovascular disease. Therefore, it is very important to identify the risk factors of NAFLD for the development of new preventive or therapeutic strategies.

NAFLD is considered the hepatic manifestation of metabolic syndrome, which is associated with insulin resistance.^2^ Metabolic disorders, such as hypertension, hyperlipidemia, diabetes, and central obesity, are known risk factors of NAFLD. Thyroid hormones regulate various metabolic processes involving carbohydrates, lipids, and proteins. Thyroid hormones also play important roles in hepatic lipid metabolism and hepatic insulin resistance. Hypothyroidism is associated with reduced lipolysis and decreased liver uptake of free fatty acids derived from triglycerides.^3^ In recent years, the correlation between overt or subclinical hypothyroidism and NAFLD has been discussed and is considered controversial.^4^, ^5^, ^6^, ^7^, ^8^ In addition, the relationship between NAFLD and thyroid function parameters remains unclear. The present study aimed to determine the relationship of NAFLD with thyroid function parameters and subclinical hypothyroidism.

## Methods

### *Study population*

Between December 2006 and December 2012, 2134 patients who visited the Third Department of Internal Medicine at Nara Medical University Hospital underwent thyroid hormone measurement. The exclusion criteria were as follows: use of medications such as thyroid hormone and antithyroid drugs, excessive alcohol intake (>20 g/day), and laboratory or clinical evidence suggesting or confirming an underlying chronic liver disease (viral hepatitis, autoimmune hepatitis, or other liver disease). Of the 2134 patients, 770 were considered for inclusion. Of these 770 patients, 70 had subclinical hypothyroidism, and they were matched according to gender, age, and body mass index (BMI) with 70 controls with euthyroidism. All study patients provided written informed consent before participating in this study. This cross‐sectional study was approved by the Institutional Review Board of Nara Medical University, and the study conformed to ethical and humane principles, as well as the Helsinki declaration.

### *Clinical and laboratory assessments*

Each participant completed a medical history questionnaire, an anthropometric assessment, and laboratory tests. BMI was calculated as follows: BMI = body weight (kg)/height squared (m^2^). Laboratory tests included the following: platelet count, baseline thyroid function (thyroid‐stimulating hormone [TSH] and free thyroxine [FT4] levels), serum alanine aminotransferase (ALT), aspartate aminotransferase (AST), total cholesterol, triglyceride, high‐density lipoprotein (HDL) cholesterol, glucose, HbA1c, hepatitis B surface antigen, and hepatitis C virus antibody. All laboratory tests were performed using standard laboratory methods. The FIB‐4 index for noninvasive markers of liver fibrosis was calculated as follows: FIB‐4 index = (age [years] × AST)/(platelet count [10^9^/L] × √ALT).^9^

### *Definitions*

Subclinical hypothyroidism was defined as a serum TSH level of >4.00 μU/L with an FT4 level between 0.90 and 1.80 ng/dL. Euthyroidism was defined as a serum TSH level between 0.35 and 4.00 μU/L, with an FT4 level between 0.90 and 1.80 ng/dL. Diabetes mellitus was defined as a fasting serum glucose level ≥126 mg/dL or use of antidiabetic medication. Hypertension was defined as a systolic blood pressure >140 mmHg or diastolic blood pressure >90 mmHg or the use of antihypertensive medication. ALT and AST elevations were defined as levels >30 U/L. NAFLD was defined by the presence of liver steatosis on ultrasonography.

The prevalence of NAFLD and the proportion of liver enzyme elevation were compared between patients with euthyroidism and those with subclinical hypothyroidism. The independent predictors of NAFLD were determined by multivariate analysis. In addition, the FIB‐4 index was compared between patients with euthyroidism and those with subclinical hypothyroidism.

### *Ultrasonography*

Fatty liver was observed via ultrasonography when there was increased liver echogenicity (“bright liver”) and a contrast between hepatic and renal parenchymal tissues.^10^ Ultrasonography was performed by experienced radiologists using the LOGIQ 7, S8, E9 (GE Healthcare Waukesha, WI, USA), Aplio 500, or Xario system (Canon Medical Systems, Tochigi, Japan).

### *Statistical analysis*

Continuous variables were compared between the two groups using Student's *t*‐test or the Mann–Whitney *U* test, and categorical variables were compared using the chi‐square test. Multivariate analysis was performed according to the results of univariate analysis. All statistical analyses were performed using JMP 11.0 software (SAS Inc., Cary, NC, USA). A *P*‐value <0.05 was considered statistically significant.

## Results

### *Baseline characteristics*

The characteristics of the study population are shown in Table 1. Seventy patients were considered to have subclinical hypothyroidism. The FT4 level was not significantly different between patients with subclinical hypothyroidism and those with euthyroidism. In addition, the prevalence of diabetes or hypertension was not significantly different between patients with subclinical hypothyroidism and those with euthyroidism. Moreover, the total cholesterol, triglyceride, HDL‐cholesterol, or HbA1c level was not significantly different between patients with subclinical hypothyroidism and those with euthyroidism. The prevalence of NAFLD was significantly higher in patients with subclinical hypothyroidism than in those with euthyroidism.

**Table 1:** Characteristics of the study population according to thyroid hormone levels

Variable	Euthyroidism (*n* = 70)	Subclinical hypothyroidism (*n* = 70)
Age (years)	68.3 ± 7.3	69.1 ± 8.1
Gender, female, *n* (%)	34 (48.6)	34 (48.6)
BMI (kg/m^2^)	24.2 ± 3.7	23.6 ± 3.3
ALT (IU/L)	26.8 ± 26.1	45.5 ± 115.6
AST (IU/L)	27.4 ± 20.1	45.3 ± 88.9
Gamma‐glutamyl transferase (IU/L)	45.2 ± 45.7	104.9 ± 250.6
Total cholesterol (mg/dL)	204.7 ± 41.9	197.1 ± 42.2
Triglyceride (mg/dL)	132.9 ± 76.5	133.6 ± 95.7
HDL‐cholesterol (mg/dL)	58.0 ± 16.8	56.0 ± 18.6
HbA1c (%)	6.5 ± 1.7	6.1 ± 1.3
Diabetes mellitus, *n* (%)	36 (51.4)	32 (45.7)
Hypertension, *n* (%)	39 (55.7)	39 (55.7)
FT4 (ng/dL)	1.23 ± 0.16	1.16 ± 0.19
TSH (μU/L)	2.20 ± 1.05	6.02 ± 213**
NAFLD, *n* (%)	10 (14.3)	24 (34.3)**
ALT elevation, *n* (%)	18 (25.7)	27 (38.6)
AST elevation, *n* (%)	16 (22.9)	26 (37.1)

** *P* < 0.01 euthyroidism *versus* subclinical hypothyroidism.

Quantitative variables are presented as mean ± standard deviation or median (interquartile range).

ALT, alanine aminotransferase; AST, aspartate aminotransferase; BMI, body mass index; FT4, free thyroxine; HDL, high‐density lipoprotein; NAFLD, non‐alcoholic fatty liver disease; TSH, thyroid‐stimulating hormone.

### *Subclinical hypothyroidism was an independent risk factor of NAFLD*

NAFLD was significantly associated with subclinical hypothyroidism, age, gender, and BMI as assessed by univariate analysis. Moreover, subclinical hypothyroidism was independently associated with NAFLD adjusted by metabolic‐related factors, such as BMI, triglyceride, HDL‐cholesterol, hypertension, and diabetes, as assessed by multivariate analysis (Table 2).

**Table 2:** Risk factors of non‐alcoholic fatty liver disease assessed by univariate and multivariate analyses

	Univariate model			Multivariate model		
Variable	OR	95% CI	*P*‐value	OR	95% CI	*P*‐value
Subclinical hypothyroidism	3.13	1.40–7.47	0.005	4.74	10.91–12.91	0.001
Age	0.91	0.86–0.96	0.001	0.91	0.85–0.97	0.002
Gender	2.39	1.09–5.47	0.03	1.62	0.66–4.09	0.295
BMI	1.17	1.05–1.33	0.006	1.2	1.05–1.39	0.009
Triglyceride	0.98	0.96–1.00	0.148			
HDL‐cholesterol	0.98	0.96–1.01	0.148			
Hypertension	1.36	0.62–3.06	0.443			
Diabetes	1.7	0.78–3.77	0.183			

BMI, body mass index; CI, confidence interval; HDL, high‐density lipoprotein; OR, odds ratio.

### *TSH elevation was an independent risk factor of NAFLD*

TSH levels were independently associated with NAFLD, adjusted by metabolic‐related factors such as BMI, triglyceride, HDL‐cholesterol, hypertension, and diabetes, as assessed by multivariate analysis. However, FT4 levels were not significantly associated with NAFLD, even in the univariate analysis (Table 3).

**Table 3:** Association between non‐alcoholic fatty liver disease and thyroid function parameters assessed by univariate and multivariate analyses

	Univariate model			Multivariate model		
Variable	OR	95% CI	*P*‐value	OR	95% CI	*P*‐value
FT4	0.12	0.01–1.31	0.083			
TSH	1.2	1.03–1.40	0.016	1.12	1.01–1.40	0.033
Gender	2.39	1.09–5.47	0.03	1.95	0.85–4.60	0.117
BMI	1.17	1.05–1.33	0.006	1.15	1.02–1.30	0.025
Triglyceride	0.98	0.96–1.00	0.148			
HDL‐cholesterol	0.98	0.96–1.01	0.148			
Hypertension	1.36	0.62–3.06	0.443			
Diabetes	1.7	0.78–3.77	0.183			

BMI, body mass index; CI, confidence interval; FT4, free thyroxine; HDL, high‐density lipoprotein; OR, odds ratio; TSH, thyroid‐stimulating hormone.

### *TSH elevation could be associated with the progression of liver fibrosis*

**Figure 1:**
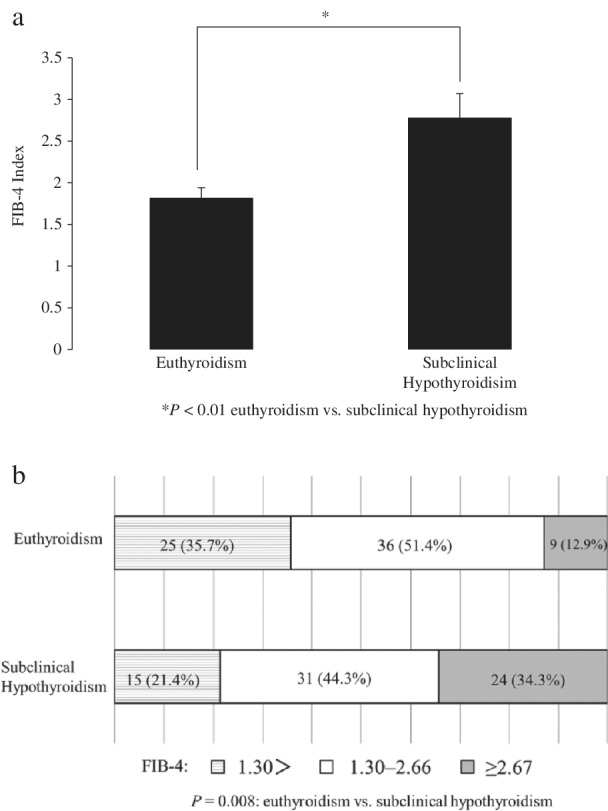
(a) The FIB‐4 index, a noninvasive marker of liver fibrosis was significantly higher in patients with subclinical hypothyroidism than in those with euthyroidism. (b) The proportion of FIB‐4 index <1.30, 1.30–2.66, and ≥2.67.

The FIB‐4 index was significantly higher in patients with subclinical hypothyroidism than in those with euthyroidism (Fig. 1a). Compared with patients with euthyroidism, the proportion of the FIB‐4 index (≥2.67) was significantly higher and the proportion of FIB‐4 index <1.30 was lower in patients with subclinical hypothyroidism (*P* = 0.008) (Fig. 1b).

## Discussion

The present study showed that TSH elevation within the normal clinical range of FT4 is an independent risk factor of NAFLD and might be associated with liver fibrosis. The prevalence of NAFLD was significantly higher in patients with subclinical hypothyroidism than in those with euthyroidism. TSH was independently associated with NAFLD in multivariate analysis, but FT4 was not an independent risk factor of NAFLD. Furthermore, the FIB‐4 index was significantly higher in patients with subclinical hypothyroidism than in those with euthyroidism.

Thyroid hormones act as potent regulators of metabolic and energy homeostasis and have been implicated in various metabolic diseases. Hypothyroidism reduces resting energy expenditure, lipolysis, and gluconeogenesis; increases weight; and increases cholesterol levels. Therefore, hypothyroidism leads to hyperlipidemia, obesity, and insulin resistance, which are risk factors of the metabolic syndrome associated with NAFLD. However, recent meta‐analyses investigating the association of hypothyroidism with NAFLD showed inconsistent results.^4^, ^11^, ^12^ Jaruvongvanich *et al*. have reported that NAFLD is not associated with thyroid hormone levels and hypothyroidism.^4^ Conversely, results of other meta‐analyses have indicated that there is an association between NAFLD and hypothyroidism.^11^, ^12^ Based on the results of those meta‐analyses, the relationship between NAFLD and thyroid function parameters is controversial. Guo *et al*. have reported that the association between NAFLD and FT3 and FT4 levels was heterogeneous among the population, and the TSH level may be an important risk factor for the development and progression of NAFLD, independent of thyroid hormones. He *et al*. have reported that the correlation between overt hypothyroidism and NAFLD was more significant than that between subclinical hypothyroidism and NAFLD. Mantovani *et al*. reported that subclinical hypothyroidism was not independently associated with the risk of incident NAFLD.^13^ However, Chung *et al*. reported a positive association between NAFLD and TSH.^7^ They showed that subclinical hypothyroidism was closely related to NAFLD in a TSH dose‐dependent manner, even within the normal upper TSH level range. In addition, Kim *et al*. reported that an increase in the TSH level, even within the normal clinical range of T4, was associated with biopsy‐proven non‐alcoholic steatohepatitis (NASH) and advanced fibrosis.^14^ The present study also showed that subclinical hypothyroidism and the TSH level were significantly related to NAFLD, but FT4 was not an independent risk factor of NAFLD.

Thyroid hormones are known to increase hepatic lipogenesis and enhance β‐oxidation.^15^ Therefore, they influence hepatic fat accumulation. High TSH levels are considered to be associated with metabolic abnormalities, as insulin resistance found in patients with clinical and subclinical hypothyroidism.^16^, ^17^ In our study, the association between TSH and NAFLD was significant after adjustment for BMI, triglyceride, HDL‐cholesterol, hypertension, and diabetes. Although the mechanism cannot be explained by our study, our findings indicate that TSH might directly contribute to the development of NAFLD. Recently, multiple studies reported extra‐thyroid tissues expressing the TSH receptor.^18^ It has been reported that functional TSH receptors were expressed in hepatocytes and that the cAMP/PKA/CREB pathway of the liver was involved in the induction of cholesterol synthesis by TSH.^19^, ^20^

The FIB‐4 index was higher in patients with subclinical hypothyroidism than in those with euthyroidism. TSH might influence the progression of liver fibrosis. Because Lee *et al*. showed a significant relationship between the level of TSH and FGF21, FGF21 might be related to the progression of liver fibrosis involving TSH.^21^ Thyroid hormones could control the development of fibrosis through an effect on adiponectin regulation.^22^, ^23^ Kowalska *et al*. demonstrated that, after L‐thyroxine treatment, insulin sensitivity significantly improved in patients with subclinical hypothyroidism.^17^ Therefore, L‐thyroxine treatment in patients with subclinical hypothyroidism might have a beneficial effect on NAFLD and liver fibrosis. Kim *et al*. reported that subclinical hypothyroidism is an independent predictor of histological NASH and advanced fibrosis.^14^ Their study was limited by biopsy‐proven NASH subjects. The subjects included in our study were outpatients in our department, which is a department of gastroenterology, endocrinology, and metabolism. Although we did not perform liver biopsy, the FIB‐4 index is the most useful marker in differentiating patients with advanced fibrosis.^24^ If the FIB‐4 index was <1.30, the possibility of NASH with advanced fibrosis was excluded, and if it was ≥2.67, the possibility of NASH with advanced fibrosis was highly suspected. In our study, the proportion of FIB‐4 index ≥2.67 was significantly higher and the proportion of FIB‐4 index <1.30 was lower in patients with subclinical hypothyroidism compared with patients with euthyroidism.

The present study has some limitations. First, this was a cross‐sectional study. Second, as NAFLD was diagnosed by ultrasonography, there was limited accuracy for the detection of mild steatosis. Third, our study included only Japanese patients who visited our department.

In conclusion, TSH is an independent risk factor of NAFLD and might have an influence on the progression of liver fibrosis.
